# Supplementary material for: Unfavorable Outcomes and Their Risk Factors in Hospitalized Patients with Staphylococcus aureus Bacteremia in the US: A Multicenter Retrospective Cohort Study, 2020–2022
Source: Antibiotics (Basel). 2025 Mar 20;14(3):326. doi: 10.3390/antibiotics14030326 (PMC11939448; doi:10.3390/antibiotics14030326)
Supplement: Supplementary file 1 [file antibiotics-14-00326-s001.zip › antibiotics-3497311-supplementary.pdf]

Supplemental Material

File S1

Definitions of SAB types

- MRSA and MSSA
    - MRSA will be identified as
        - Organism listed as MRSA, or
        - A SA isolate that is NOT susceptible to oxacillin or methicillin or nafcillin or cefazolin
- Uncomplicated and Complicated
    - Complicated SAB will be defined as
        - Persistent positive blood culture for SA (concordance between index and follow-up organism – MSSA and MRSA – will be required) on day 2, 3 or 4 following index culture
        - H/o hemodialysis (ICD-10 codes 5A1D00Z, 5A1D60Z, 3E1M39Z, or CPT code 90935)
        - Secondary to (based on culture [obtained within the range of 3 days prior to 3 days following index culture] positive for the same organism as the BSI, from the respective sites [except for endocarditis – see below]):
            - Skin and soft tissue infection (SSTI)
            - Joint
            - Bone
            - Vascular
            - Cerebrospinal fluid (CSF)

        - Other central nervous system (CNS)
        - Heart
        - Lung
        - Pleura
        - Endocarditis (as evidenced by ICD-10 code I33.0 AND evidence of a transesophageal echocardiogram [transesophageal echocardiogram (TEE), CPT codes 93312-93318, 93355] obtained within 5 days of index culture)
      - TEE [CPT codes 93312-93318, 93355] obtained within 5 days of index culture
    - Related vs. unrelated to a central line (i.e., central line-associated BSI [CLABSI] vs. not CLABSI)
      - CLABSI defined as a central venous catheter (CVC) placement within 5 days prior to index culture date (as evidenced by a CPT code 36488-36491 or a charge code) OR a CVC tip positive for the same organism as the index blood culture
- Community- (CA) and hospital-acquired (HA)
    - Differentiated based on the timing of the index culture
      - If within 3 days of admission, then CA
      - If on day 4 of admission or later, then HA

File S2

Elixhauser comorbidities

	Good	%	Poor	%	P-value

	N = 1653		N = 2427		
Acquired immune deficiency syndrome	16	0.97%	35	1.44%	0.181
Alcohol abuse	121	7.32%	219	9.02%	0.053
Deficiency anemias	546	33.03%	1133	46.68%	<0.001
Autoimmune conditions	83	5.02%	159	6.55%	0.042
Chronic blood loss anemia	21	1.27%	42	1.73%	0.242
Leukemia	15	0.91%	28	1.15%	0.450
Lymphoma	15	0.91%	39	1.61%	0.055
Metastatic cancer	64	3.87%	72	2.97%	0.114
Solid tumor without metastasis, in situ	0	0.00%	0	0.00%	1.000
Solid tumor without metastasis, malignant	56	3.39%	78	3.21%	0.760
Cerebrovascular disease	85	5.14%	189	7.79%	0.001
Coagulopathy	199	12.04%	492	20.27%	<0.001
Dementia	153	9.26%	151	6.22%	<0.001
Depression	298	18.03%	483	19.90%	0.135
Diabetes with chronic complications	599	36.24%	981	40.42%	0.007
Diabetes without chronic complications	135	8.17%	141	5.81%	0.003
Drug abuse	240	14.52%	453	18.67%	0.001
Heart failure	381	23.05%	696	28.68%	<0.001
Hypertension, complicated	535	32.37%	1017	41.90%	<0.001
Hypertension, uncomplicated	613	37.08%	754	31.07%	<0.001
Liver disease, mild	189	11.43%	395	16.28%	<0.001
Liver disease, moderate to severe	43	2.60%	95	3.91%	0.023
Chronic pulmonary disease	362	21.90%	575	23.69%	0.182
Neurological disorders affecting movement	40	2.42%	73	3.01%	0.261
Other neurological disorders	211	12.76%	483	19.90%	<0.001
Seizures and epilepsy	75	4.54%	121	4.99%	0.511
Obesity	463	28.01%	629	25.92%	0.138
Paralysis	84	5.08%	161	6.63%	0.041
Peripheral vascular disease	154	9.32%	314	12.94%	<0.001
Psychoses	113	6.84%	175	7.21%	0.647
Pulmonary circulation disease	87	5.26%	176	7.25%	0.011
Renal failure, moderate	291	17.60%	414	17.06%	0.650
Renal failure, severe	74	4.48%	340	14.01%	<0.001

Hypothyroidism	258	15.61%	353	14.54%	0.350
Other thyroid disorders	26	1.57%	27	1.11%	0.202
Peptic ulcer disease with bleeding	30	1.81%	58	2.39%	0.215
Valvular disease	306	18.51%	622	25.63%	<0.001
Weight loss	163	9.86%	373	15.37%	<0.001

File S3

Antimicrobial escalation

	**Unfavorable Outcomes (N=2,427)**	
	N	%
Switch from vancomycin to daptomycin	590	24.31%
Switch from vancomycin to ceftaroline	212	8.74%
Switch from daptomycin to ceftaroline	42	1.73%
Switch from vancomycin to daptomycin & ceftaroline	151	6.22%
Addition of ceftaroline to daptomycin	240	9.89%
Addition of aminoglycoside or fluoroquinolone to vancomycin	231	9.52%
Addition of aminoglycoside or fluoroquinolone to daptomycin	31	1.28%
